# Familial Essential Thrombocythemia Associated with *MPL* W515L Mutation in Father and *JAK2* V617F Mutation in Daughter

**DOI:** 10.1155/2014/841787

**Published:** 2014-11-10

**Authors:** Adrian P. Trifa, Andrei Cucuianu, Radu A. Popp

**Affiliations:** ^1^Department of Medical Genetics, “Iuliu Hatieganu” University of Medicine and Pharmacy, 400349 Cluj-Napoca, Romania; ^2^Department of Haematology, “Ion Chiricuta” Cancer Institute, Cluj-Napoca, Romania

## Abstract

Familial essential thrombocythemia features the acquisition of somatic mutations and an evolution similar to the sporadic form of the disease. Here we report two patients—father and daughter—with essential thrombocythemia who displayed a heterogeneous pattern of somatic mutations. The *JAK2* V617F mutation was found in the daughter, while the father harbored the *MPL* W515L mutation. This case report may constitute further proof that in familial essential thrombocythemia there are other, still undefined, constitutional, inherited genetic factors predisposing to the acquisition of various somatic mutations (e.g., *JAK2* V617F and *MPL*).

## 1. Introduction

Essential thrombocythemia (ET) is characterized in about half of the cases by the somatic mutation* JAK2* V617F. Around 5–10% of the* JAK2*-negative patients harbor somatic mutations within the* MPL* gene. Finally, up to 80% of the* JAK2*-negative patients harbor somatic mutations within the* CALR* gene [1].

Familial ET resembles sporadic ET in terms of clinical presentation and acquisition of somatic mutations. It is known that there is a genetic predisposition for myeloproliferative neoplasms (MPN) in general, including ET, but there is no proven monogenic defect to date. Familial ET must not be confused with hereditary thrombocytosis. Although some patients harbor germline mutations within the* THPO* (thrombopoietin) or* MPL* (thrombopoietin receptor) genes, the molecular defect still remains unknown in most instances of hereditary thrombocytosis. It is of note that hereditary thrombocytosis usually features a classical Mendelian transmission. Unlike ET, hereditary thrombocytosis is not a true clonal disease [2].

A large Swedish epidemiological study revealed for the first time a 12-fold increased risk of developing ET among the first-degree relatives of patients with ET [3]. Pardanani et al. reported an association between several SNPs within the* JAK2* locus and PV (polycythemia vera) and ET [4]. Shortly after, the* JAK2* 46/1 haplotype was demonstrated as the most important common constitutional genetic factor predisposing to the acquisition of the* JAK2* V617F mutation. According to Jones et al.,* JAK2* 46/1 predisposes not only to* JAK2*-mutated MPN, but also to* MPL*-mutated MPN [5]. However, half of the patients with ET lack the* JAK2* V617F or* MPL* mutations. Thus, other polymorphisms or mutations not yet discovered should be responsible for the occurrence of the familial ET. More recently, the C allele of the rs2736100 SNP, located in the* TERT* gene, was identified as a strong susceptibility factor for MPN, initially in the Icelandic population, and then confirmed in a large cohort of Italian patients with MPN [6, 7]. This SNP correlated with all molecular and clinical MPN subtypes, independently of* JAK2* 46/1 haplotype. Together with the* JAK2* 46/1 haplotype, the rs2736100 SNP explained 73.06% of the population attributable fraction. Interestingly, the rs2736100 SNP was significantly enriched in familial MPN compared to sporadic MPN, suggesting a role of this SNP in familial clustering of the MPN [7]. Here we report two patients with ET, in which we found the* JAK2* V617F mutation (in the daughter) and the* MPL* W515L (in the father).

## 2. Case Report

B. L., a 43-year-old Caucasian female, was referred in 2007 to our service for thrombocytosis (1,000 × 10^9^/L), discovered during a routine blood checkup. The patient was asymptomatic and there was no personal or familial history of thrombosis or hemorrhage. The full blood counts also revealed a mild leukocytosis (12 × 10^9^/L), with a normal differential count, except for mild basophilia (0.24 × 10^9^/L). The hemoglobin and the hematocrit were within normal range (140 g/L and 42%, resp.). The physical examination revealed no pathologic modifications. A bone marrow biopsy was also performed, revealing megakaryocyte hyperplasia. Thus, the diagnosis of ET was established. In 2008 the detection of the* JAK2* V617F mutation became available in our institution, and a mutant allele burden around 25% was found in the patient. The patient was given initially acetylsalicylic acid (ASA) 150 mg/day. However, because the platelets continued to increase to values of around 1,500 × 10^9^/L, the patient was subsequently started on anagrelide, 2 mg/day. Thereafter, the platelets dropped to normal values, with the patient remaining asymptomatic.

Four years later, the father of the patient, P. T., a 72-year-old man, was referred to our service for thrombocytosis (1,100 × 10^9^/L), discovered during a routine blood checkup. The blood counts and the peripheral smear revealed no other modifications. The physical examination was normal. The* JAK2* V617F mutation tested negative at diagnosis, but other causes of thrombocytosis were ruled out. Thus, the diagnosis of ET was established. The patient was given 150 mg ASA and 1 g hydroxyurea/day. The platelets dropped to near normal values, with the patient remaining asymptomatic. Later, the diagnosis of* MPL* exon 10 and* CALR* exon 9 mutations became available in our institution. The patient lacked the* CALR* mutations, but he was found to harbor the* MPL* W515L mutation.

We genotyped in both patients also the rs10974944 SNP, which tags the* JAK2* 46/1 haplotype. Both of them were homozygous for the “nonrisk” C allele of the rs10974944 SNP. Thus, none of them carried the* JAK2* 46/1 haplotype.

All the genotyping procedures were performed on DNA obtained from peripheral blood, using a commercially available kit (Quick-gDNA MiniPrep Kit, ZymoResearch, Irvine, CA, USA). The* JAK2* V617F and* MPL* mutations were genotyped by tetraprimer PCR and multiplex PCR assays, as previously described [8, 9]. The rs10974944 SNP, tagging the* JAK2* 46/1 haplotype, was genotyped by a PCR-RFLP assay, as we described previously [10]. In order to analyze the common mutations within the exon 9 of the* CALR* gene (type 1 and type 2 mutations), which account for almost 90% of all* CALR* mutations, we developed a simplex PCR assay. We analyzed the sequence of* CALR* gene and developed primers encompassing the two common mutations, using the online program Primer3 v.0.4.0 [11]. The primers chosen have the following sequences: forward 5′-GCAGCAGAGAAACAAATGAAGG-3′ and reverse 5′-CTTCCTCCTTGTCCTCCTCA-3′. The reactions were set up in 20 *μ*L volumes, with the following composition: 10 *μ*L 2xPCR MasterMix (Fermentas MBI, Vilnius, Lithuania), 10 pmoles of each Fw and Rev primers, 1 *μ*L of 2 mg/mL BSA solution (Fermentas MBI, Vilnius, Lithuania), and 50 ng of genomic DNA. The amplifications were run in Applied Biosystems 2720 and Eppendorf Mastercycler thermal cyclers (Applied Biosystems, USA and Eppendorf, Germany), under the following conditions: initial denaturation at 95°C for 10 min, followed by 35 cycles, each comprising denaturation at 95°C for 40 s, annealing at 55°C for 45 s, and extension at 72°C for 40 s, followed by a final extension at 72°C for 5 min. The amplicons were resolved in a 4% MetaPhor agarose gel (Lonza, Rockland, USA), stained with RedSafe (Chembio, UK). In order to validate our PCR assay, we randomly selected 24 samples and sequenced them bidirectionally, using the same primers as described above. There was a 100% concordance between the results obtained by sequencing and those obtained by the PCR assay.

The study was carried out according to the guidelines of the Declaration of Helsinki and informed consent was obtained from both patients.

We also performed a routine clinical and hematological examination on Ms. B. L.'s two daughters and on Mr. P. T.'s sister, but there were no pathological aspects that would suggest the presence of MPN. In order to search for a possible latent MPN, we also genotyped* JAK2* V617F,* CALR*, and* MPL* mutations in these three relatives of the patients, but none of them harbored any of these mutations.

During follow-up, both patients had a favorable evolution, and to date none of them had any of the ET-associated complications (thrombosis or progression to myelofibrosis).

## 3. Discussion

We describe two patients with ET—father and daughter—in which we found the* MPL* W515L mutation in the father and the* JAK2* V617F mutation in the daughter. This suggests the presence of a constitutional genetic factor in both of them. The* JAK2* 46/1 haplotype is a known constitutional genetic factor strongly associated with* JAK2* V617F-positive MPN. It was expected that this haplotype might explain at least in part the genetic predisposition seen in familial MPN. Olcaydu et al. analyzed 88 familial MPN patients from 52 families and 772 apparently sporadic MPN patients [12]. They reported the* JAK2* 46/1 haplotype was associated with the occurrence of the* JAK2* V617F mutation in the familial cases (*P* value = 4.929 × 10^−6^) in the same manner as in the sporadic cases (*P* value = 7.19 × 10^−13^) but did not underlie the familial clustering of the diseases (*P* value = 0.6529). In fact, the authors calculated the penetrance of the* JAK2* 46/1 haplotype and they found it was only about 0.02%, while the penetrance in familial MPN was around 31–35% [12]. The authors also described a family with three affected members, in which they found the* JAK2* V617F mutation in a primary myelofibrosis (PMF) patient and the* MPL* W515L in an ET patient. The third patient of this family diagnosed with a MPN (PMF) did not harbor* JAK2* or* MPL* mutations but carried deletions on chromosomes 2p, 7q, and 15q [12]. The* JAK2*-positive patient was heterozygous for the* JAK2* 46/1 haplotype, while the* MPL*-positive patient was homozygous for the “nonrisk” haplotype. Thus, the* JAK2* 46/1 haplotype did not segregate with the MPN phenotype in this family [12]. On the other hand, our patients were both homozygous for the C allele (“nonrisk” allele) of the rs10974944 SNP tagging the* JAK2* 46/1 haplotype, so we may exclude this factor as a contributor to the occurrence of the disease in our family.

In the past, it was more difficult to diagnose familial MPN, since the medical records were not available for all the members; also some affected members were perhaps asymptomatic and the molecular markers specific for these diseases had not yet been discovered. It is estimated that around 5–10% of sporadic MPN cases represent in fact familial MPN [2]. This mirrors the observations of the large Swedish epidemiological study, which reported an increased risk of developing a MPN among first-degree relatives of affected patients. This is true not only for ET, but for all MPN [3]. In fact, around 60% of the familial MPN cases show a homogeneous disease subtype; the remainder show a heterogeneous pattern in the distribution of MPN subtypes [2]. On the other hand, we were not able to find other relatives affected by a MPN in the currently reported family.

Malak et al. reported on a large cohort of 93 families with MPN, comprising 227 patients, of which 105 had ET. Among these 105 patients, 58 (60%) had the* JAK2* V617F mutation, which is a similar figure as in the sporadic cases [13]. The authors provided a long term follow-up of this cohort of patients and they concluded the overall prognosis of the familial ET is similar to the sporadic form of the disease. Langabeer et al. analyzed the* JAK2* V617F,* CALR*, and* MPL* mutations in affected members of 9 familial MPN series and a family affected rather by hereditary thrombocytosis. Across the 9 families, there were 13 individuals affected of ET, but the molecular analysis was unavailable in four of them.* JAK2* V617F mutation was seen in three patients, while the* CALR* type 1 mutation was detected in two of them. No patient was found to harbor* MPL* mutations [1]. Maffioli et al. analyzed 10 families with at least two members with MPN, comprising 21 affected patients: 15 with ET, 3 with PV, one with prefibrotic PMF, and 2 with chronic myeloid leukemia. Nineteen of the 21 affected patients had a known status for* JAK2* V617F: 13 of them (68%) were* JAK2*-positive. Among the 6* JAK2*-negative patients, there were two with* CALR* mutations: one with ET and the other with prefibrotic PMF [14]. Thus, the distribution of the somatic mutations in these series of familial MPN was similar to that found in the sporadic cases.

In conclusion, our report demonstrated once again that affected individuals in familial ET show a heterogeneous spectrum of somatic mutations (*JAK2* V617F and* MPL* W515L in our case). This pattern is similar to the sporadic form of the disease. The genetic factors predisposing to familial ET are yet to be discovered.
